# Mutual Information in Conjugate Spaces for Neutral Atoms and Ions

**DOI:** 10.3390/e24020233

**Published:** 2022-02-02

**Authors:** Juan Carlos Angulo, Sheila López-Rosa

**Affiliations:** 1 Departamento de Física Atómica, Molecular y Nuclear, Universidad de Granada, 18010 Granada, Spain; 2 Instituto Carlos I de Física Teórica y Computacional, Universidad de Granada, 18010 Granada, Spain; slopezrosa@us.es; 3 Departamento de Física Aplicada II, Universidad de Sevilla, 41004 Sevilla, Spain

**Keywords:** mutual information, quantum similarity index, electron correlation, multielectronic systems

## Abstract

The discrepancy among one-electron and two-electron densities for diverse *N*-electron atomss, enclosing neutral systems (with nuclear charge Z=N) and charge-one ions (|N−Z|=1), is quantified by means of mutual information, *I*, and Quantum Similarity Index, QSI, in the conjugate spaces position/momentum. These differences can be interpreted as a measure of the electron correlation of the system. The analysis is carried out by considering systems with a nuclear charge up to Z=103 and singly charged ions (cations and anions) as far as N=54. The interelectronic correlation, for any given system, is quantified through the comparison of its double-variable electron pair density and the product of the respective one-particle densities. An in-depth study along the Periodic Table reveals the importance, far beyond the weight of the systems considered, of their shell structure.

## 1. Introduction

In any atomic system, the probability of finding an electron in a determinate region is closely related to the value of one-electron density. Studying the atomic density in different regions allows us to understand how electrons belonging to specific regions behave. In that sense, monoelectronic densities $\rho (\vec{r})$ contain an enormous amount of encoded information. In fact, $\rho (\vec{r})$ determines the physical and chemical properties of atoms and molecules, as it is well known [1]. Nevertheless, information on interelectronic correlation is absent from the one-electron density. Thus, $\rho (\vec{r})$ does not provide any information on the eventual influence of the position of a given electron on those of the others. It is precisely in this framework where two-electron densities arise. The electron pair density $\Gamma ({\vec{r}}_{1},{\vec{r}}_{2})$ quantifies the probability of finding two arbitrary electrons located at positions ${\vec{r}}_{1}$ and ${\vec{r}}_{2}$, respectively, and constitutes an appropriate starting point to analyze explicitly the spatial electron–electron interaction [2]. Moreover, in quantum chemistry, the pair density allows describing the quantum-mechanical distribution of electron pairs so that it constitutes a fundamental tool for studying chemical binding [3] as well as electronic correlation and structure in molecules [4,5].

Moreover, the electron pair density determines the exact interaction energy of a many-electron system, while the standard Kohn–Sham density functional models do not approximate it properly [6,7]. Therefore, pair density functional theory is considered as both an extension of the density functional theory and a reduced density matrix theory [8,9]. The main point of the pair density functional theory lies in its fundamental variable, namely the pair density, more informative than the monoelectronic density regarding the electron–electron interaction [10,11].

The electron pair density allows obtaining the expectation value of any arbitrary two-particle operator. Moreover, the pair density functional theory has another virtue, related to the expansion of the approximate functional: the exchange-correlation energy functional is rigorously expressed in terms of the pair density so that the only need is to select properly the approximate form of the kinetic energy functional [12]. The recent development of multiconfiguration pair density functional theory combines the advantages of both wave function and density functional theories, thus improving the handling of strongly correlated systems. The results obtained are comparable to those of some Kohn–Sham density functionals, including both traditional and modern ones [13,14].

Consequently with the above, it appears logical to conjecture that electron pair densities for many-electron systems have been studied in depth, both in intensive and extensive ways. However, that is not the case: More efforts have been devoted to analyses in terms of one-electron densities, compared to those based on electron pair densities. It is worth noting that, despite the vast amount of information encoded in monoelectronic densities, they do not provide any clues on how the location of an electron in position/momentum space constrains the respective locations of the others. Let us also highlight that within the Waller–Hartree theory, the two-electron density is related to total X-ray scattering intensity, an experimentally accessible quantity [15,16].

A relevant difficulty to handle the electron pair density, a function of six coordinates, is its spatial interpretation. Normally, the visual representation of a distribution over its domain constitutes a powerful analytical tool, but this is not a feasible procedure with such a number of variables. In order to extract useful information from the electron pair density, previous handlings must be performed. Particularly interesting are those giving rise to so-called intracule and extracule distributions, with a huge variety of well-known results based on them [17,18,19,20,21]. It is worth noting that these densities consider any pair of electrons as a unit. A further simplification is achieved by spherically averaging the electron pair density to obtain a function with only radial coordinates as variables. Of course, two-electron information decreases as a consequence of dimensionality reduction.

At present, information theory of quantum systems is a compact theory that brings together a large number of concepts, some of which have roots remote in the past and with a very diverse origin, within the framework of disciplines that have followed a differentiated evolution but are interconnected, e.g., physics, mathematics, chemistry and biology. In fact, it is employed in a diversity of fields by means of measures and concepts at both classical (e.g., Shannon entropy, Fisher information, complexity) and quantum levels (e.g., von Neumann and other entanglement measures) [22,23]. Particularly for atomic and molecular systems, their entropic characterization complements the energetic one based on the wavefunction and within a density functional framework. The description of main physical and chemical properties of those systems can be achieved in terms of entropic spreading measures of the electron distribution [24,25], which quantify, for instance, uncertainty, randomness, disorder and localization. These concepts have induced the birth of a diversity of density functionals: Shannon entropy [26], Fisher information [24,27] and complexity [28] among others. All these functionals play a relevant role in the study of many-electron systems [29,30,31,32,33,34,35].

The analysis of the electron pair densities from an Information Theory point of view has attracted the attention of researchers over the past years. In particular, information theoretical measures such as Shannon-related ones [4,36,37], similarity measures [4,38,39,40] or uncertainty relationships [38] have been obtained. Moreover, disequilibrium, Shannon entropy, shape complexity and its corresponding information plane for the electron pair densities throughout the periodic table in both position and momentum spaces have been recently derived [41]. As a consequence, the applications of the electron pair densities have increased in different ways including an alternative density functional theory with electron pair density as the functional key [7,12,42]. These densities have been employed as a scaledown method to study many particle systems [6,43,44,45,46] and for the analysis and detection of chemical bonds in molecules for which its positions are directly related to regions where electrons present higher correlation [47,48].

According to the Density Functional Theory and the Hohenberg–Kohn theorem, physical and chemical properties of atomic and molecular systems are given by their electron charge densities [1]. Therefore, it looks very attractive to establish proper methods of quantifying differences among two or more systems attending to their respective distributions, and mostly regarding the higher/lower similarity among their main physicochemical properties. This is the origin of the Quantum Similarity Theory (QST) [49] that arises from the huge interest in chemical similarity in past years. Molecular modeling, quantitative structure activity relationship (QSAR) and quantum information are simple examples of such an interest [50,51]. In this sense, establishing density functionals that act as measures of “distance” or similarity among probability distributions appears very interesting as well. Different approaches have arised in probability, statistics and Information Theory with the aim of establishing quantitative dissimilarity measures among two or more probability densities in order to quantify how different these distribution are, resulting in the birth of a diversity of divergence measures. Their usefulness has been widely contrasted in the analysis of, e.g., different physical and chemical systems and processes, as described by means of meaningful and relevant distributions [29,52,53,54,55,56,57,58]. Each measure is more or less useful, firstly attending to its own properties and features but also considering the kind of problem, the specific system or process as well as the elucidation we are dealing with.

The aim of this work is to quantify the similarity and differences of electron pair distributions for correlated and uncorrelated variables are. In doing so, the Shannon entropy *S* (which provides a first quantitative notion of the spreading for a given distribution), the mutual information *I* (interpreted in terms of the Kullback–Leibler divergence) and the Quantum Similarity Index QSI (which constitutes a measure of the ‘overlap’ among distributions on their domain) are employed. In this analysis, neutral atoms and also their singly charged ions are considered.

The paper is structured as follows: In Section 2, two-particle densities for atomic systems and the entropic functionals used for this analysis (*S*, *I* and QSI) are defined and presented. Numerical computations and results are shown in Section 3. Finally, the last section briefly describes the conclusions, open problems and future work.

## 2. Electron Pairs in Atoms: Spreading and Correlation

The two-electron densities in conjugated spaces, obtained, respectively, from the *N*-electron wave function Ψ, and its Fourier transform Φ, are given by the following:

(1) $$\Gamma \left({\vec{r}}_{1},{\vec{r}}_{2}\right)=\int \Psi \left({\vec{x}}_{1},{\vec{x}}_{2},\cdots ,{\vec{x}}_{N}\right)\Psi^{*}\left({\vec{x}}_{1},{\vec{x}}_{2},\cdots ,{\vec{x}}_{N}\right)d\sigma_{1}d\sigma_{2}d{\vec{x}}_{3}\cdots d{\vec{x}}_{N}$$

in position space, and by the following is given in momentum space.

(2) $$\Pi \left({\vec{p}}_{1},{\vec{p}}_{2}\right)=\int \Phi \left({\vec{y}}_{1},{\vec{y}}_{2},\cdots ,{\vec{y}}_{N}\right)\Phi^{*}\left({\vec{y}}_{1},{\vec{y}}_{2},\cdots ,{\vec{y}}_{N}\right)d\sigma_{1}d\sigma_{2}d{\vec{y}}_{3}\cdots d{\vec{y}}_{N}$$

The variable ${\vec{x}}_{i}={\vec{r}}_{i}\sigma_{i}$ is a combined position–spin coordinate and similarly with the momentum–spin one ${\vec{y}}_{i}={\vec{p}}_{i}\sigma_{i}$. The physical meaning of these densities accounts for the probability of finding an electron within the region ${\vec{r}}_{1}d{\vec{r}}_{1}$ if there is another electron within ${\vec{r}}_{2}d{\vec{r}}_{2}$, both them with allowed and compatible states. The same applies to respective regions ${\vec{p}}_{1}d{\vec{p}}_{1}$ and ${\vec{p}}_{2}d{\vec{p}}_{2}$ in momentum space. Two-electron densities are straightforwardly related to the interelectronic correlation, since the compatibility of an electron state is determined by those of the others. Thus, these densities constitute distributional quantifiers of correlation between electrons.

The Hartree–Fock approach is considered for calculating the two-electron densities so that they become expressed for a *N*-electron system as follows [59]:

(3) $$\Gamma \left({\vec{r}}_{1},{\vec{r}}_{2}\right)=\frac{1}{N-1}\left[N\rho \left({\vec{r}}_{1}\right)\rho \left({\vec{r}}_{2}\right)-\Gamma_{x}\left({\vec{r}}_{1},{\vec{r}}_{2}\right)\right]$$

in position space, and the following in momentum space.

(4) $$\Pi \left({\vec{p}}_{1},{\vec{p}}_{2}\right)=\frac{1}{N-1}\left[N\gamma \left({\vec{p}}_{1}\right)\gamma \left({\vec{p}}_{2}\right)-\Pi_{x}\left({\vec{p}}_{1},{\vec{p}}_{2}\right)\right]$$

The functions $\rho (\vec{r_{i}})$ and $\gamma (\vec{p_{i}})$ are the respective one-electron densities, and $\Gamma_{x}\left({\vec{r}}_{1},{\vec{r}}_{2}\right)$ and $\Pi_{x}\left({\vec{p}}_{1},{\vec{p}}_{2}\right)$ denote the exchange densities in position and momentum space, respectively. Let us emphasize that the use of Hartree–Fock functions focuses the study on the Fermi correlation between same-spin electrons, derived from the antisymmetry of the wave function. This means that the term electron correlation, above mentioned, refers to the statistical correlation. It is necessary to clarify this point to avoid misleading when considering correlated systems beyond the Hartree–Fock approach (the Löwdin definition of correlation energy).

Remark that the information content of one-particle densities is lower than that of two-particle ones because they arise from the integrations $\rho \left(\vec{r_{1}}\right)=\int \Gamma \left({\vec{r}}_{1},{\vec{r}}_{2}\right)d{\vec{r}}_{2}$ and $\gamma \left(\vec{p_{1}}\right)=\int \Pi \left({\vec{p}}_{1},{\vec{p}}_{2}\right)d{\vec{p}}_{2}$. Thus, monoparticular densities are marginal distributions of the electron pair ones so that the latter cannot be obtained from the former.

The Shannon entropy, *S*, of the normalized-to-unity electron pair densities is given by the following:

(5) $$S_{\Gamma }=-\int \Gamma \left({\vec{r}}_{1},{\vec{r}}_{2}\right)\ln\Gamma \left({\vec{r}}_{1},{\vec{r}}_{2}\right)d{\vec{r}}_{1}d{\vec{r}}_{2}$$

for the position space, and the following for the momentum space.

(6) $$S_{\Pi }=-\int \Pi \left({\vec{p}}_{1},{\vec{p}}_{2}\right)\ln\Pi \left({\vec{p}}_{1},{\vec{p}}_{2}\right)d{\vec{p}}_{1}d{\vec{p}}_{2}$$

Entropy quantifies the spreading extent of the distribution, thus constituting a measure of uncertainty/delocalization. Let us note that, attending to the definitions above, the Shannon entropy could be negative. To guarantee non-negative uncertainties, sometimes it is useful to handle the so-called exponential Shannon entropy, defined as $L=e^{S}$. Such a definition of the exponential Shannon entropy is considered that way also to deal with a measure having the same dimensions as the variable involved, as the variance does (maybe the most widely used uncertainty measure).

Considering the spherically averaged forms of each the monoelectronic and the electron pair densities, higher values of the two-electron Shannon entropy can be expected, as compared to those in the one-electron case. This appears natural, taking into account the bidimensionality of the former and the monodimensionality of the latter, thus with respective higher/lower spreadings. Let us also observe the use of the past electron pair Shannon entropy for studying the corresponding distribution [4,36,38,40]. Some of those studies successfully revealed correlation effects and other atomic properties by means of two-electron Shannon entropy.

In order to perform a comparative analysis among atomic one-particle and two-particle densities from an informational theoretic point of view, i.e., to better understand the differences between these densities, it would be necessary to have at our disposal density functionals enabling us to quantify the “distance” and/or similarity among them.

The interest in designing adequate tools for the quantitative comparison of two or more systems or processes, as characterized by means of distribution functions, becomes enhanced due to the strong link between diverse information measures and many significant physical and chemical properties of the systems/processes. The term ‘distance’, within this context, refers to a measure of dissimilarity between functions, being not necessarily a true distance in a rigorous mathematical sense. Nevertheless, all these types of distances retain some of the well-known characteristic properties, in particular non-negativity, symmetry (invariance under exchange of functions) and saturation (minimal zero value only for identical distributions).

One of the pioneering global measures of dissimilarity between probability distributions is the so-called ‘relative entropy’ or Kullback–Leibler divergence (KL) [52]. It accounts for the amount of information supplied by the data for discriminating among the distributions. Being a ‘directed divergence’, it is not symmetric:

(7) $$KL\left(f,g\right)=\int f\left(\vec{x}\right)\ln\frac{f(\vec{x})}{g(\vec{x})}d\vec{x}$$

where $f(\vec{x})$ and $g(\vec{x})$ are arbitrary distributions over the same domain. The applications of KL for different procedures, such as the obtention of minimum cross entropy estimations or the determination of atomic and molecular properties [31], among others, cause it to be set as an information-theoretic fundamental tool.

This measure could be used to quantify differences between atomic one-particle and two-particle densities. If we compare the two-electron density with the product of the monoparticular densities, this magnitude could be interpreted as a measure of the correlation between variables [29]. It is called Mutual Information, *I*, and has been introduced into quantum chemistry [60]:

(8) $$I_{r}=\int \Gamma \left(\vec{r_{1}},\vec{r_{2}}\right)\ln\frac{\Gamma (\vec{r_{1}},\vec{r_{2}})}{\rho \left(\vec{r_{1}}\right)\rho \left(\vec{r_{2}}\right)}d\vec{r_{1}}d\vec{r_{2}}$$

for the position space, and the following for the momentum space.

(9) $$I_{p}=\int \Pi \left(\vec{p_{1}},\vec{p_{2}}\right)\ln\frac{\Pi (\vec{p_{1}},\vec{p_{2}})}{\gamma \left(\vec{p_{1}}\right)\gamma \left(\vec{p_{2}}\right)}d\vec{p_{1}}d\vec{p_{2}}$$

A recent study of the authors [61] have considered the Jensen–Shannon divergence (JSD) as a comparative measure with similar purposes to those presented here. In that case, the measure employed is a symmetrized version of KL, thus loosing the ‘directed character’ which makes KL an appropriate measure of mutual information, in the sense that it takes the uncorrelated distribution as the reference/a priori one.

Quantum Similarity Theory (QST) [49] was originally developed in order to establish quantitative comparisons between molecular systems by means of their fundamental structural magnitudes (i.e., electron density functions), and the Quantum Similarity Index (QSI) is its principal measure. This quantity provides a measure of the overlapping among the distributions under consideration. Using it to perform a comparative analysis among atomic one-particle and two-particle densities, QSI is given by the following:

(10) $$QSI_{r}=\frac{\int \Gamma \left(\vec{r_{1}},\vec{r_{2}}\right)\rho \left(\vec{r_{1}}\right)\rho \left(\vec{r_{2}}\right)d\vec{r_{1}}d\vec{r_{2}}}{\sqrt{\int \Gamma^{2}\left(\vec{r_{1}},\vec{r_{2}}\right)d\vec{r_{1}}d\vec{r_{2}}\int \rho^{2}\left(\vec{r_{1}}\right)\rho^{2}\left(\vec{r_{2}}\right)d\vec{r_{1}}d\vec{r_{2}}}}$$

for the position space, and the following for the momentum space.

(11) $$QSI_{p}=\frac{\int \Pi \left(\vec{p_{1}},\vec{p_{2}}\right)\gamma \left(\vec{p_{1}}\right)\gamma \left(\vec{p_{2}}\right)d\vec{p_{1}}d\vec{p_{2}}}{\sqrt{\int \Pi^{2}\left(\vec{p_{1}},\vec{p_{2}}\right)d\vec{p_{1}}d\vec{p_{2}}\int \gamma^{2}\left(\vec{p_{1}}\right)\gamma^{2}\left(\vec{p_{2}}\right)d\vec{p_{1}}d\vec{p_{2}}}}$$

Since QSI is also a non-negative and symmetric measure, its main characteristic feature is the upper-bounded property. The inequality QSI(f,g)≤1 holds for arbitrary pairs of functions, with the equality QSI=1 reached only in the case of identical distributions.

## 3. Entropy and Comparative Measures

Three different measures are here considered for the informational-theoretic study of atomic systems at the one-particle and two-particle levels. The Shannon entropy provides a first quantitative notion of the spreading for a given distribution.

Then, two comparative functionals are employed to quantify, for a given system, how similar/different the electron pair distributions for correlated and uncorrelated variables are. These functionals are as follows: the mutual information *I* (interpreted in terms of the Kullback–Leibler divergence) and the Quantum Similarity Index QSI (as measure of the ‘overlap’ among distributions on their domain).

The entire next analysis is two-sided. On the one hand, parallel studies are carried out by considering both conjugate spaces, namely position and momentum. On the other, not only neutral atoms are analyzed (i.e., with identical values of their nuclear charge *Z* and the number of electrons *N* as far as N=Z=103) but also their singly charged ions up to N=54.

The results here provided, and their interpretation as well, are compared with similar ones found in the literature. Main differences are related, normally, to the set of collected systems and/or the quality of wavefunctions to describe them, as well as the employment of some information-theoretic functionals at the two-particle level.

All calculations were performed, in the present work, by means of accurate and well known near Hartree–Fock wavefunctions [62,63]. Thus, all computations are performed here at a non-relativistic level, with future work planned by considering also relativistic wavefunctions. The authors have recent works with quantitative informational measures of relativistic effects in atomic systems at the one-electron level [64,65]. Those measures include mutual information [64] and a generalized version of quantum similarity [65]. The present work focuses on interelectronic correlation far beyond relativistic effects.

The study below is limited to ground-state distributions, with additional analyses of excited systems planned as future work. In this sense, it is worthy to remark that Koga’s wavefuntions here employed correspond to experimental ground states, as emphasized in the corresponding references.

### 3.1. Shannon Entropy

Previous studies have considered the Shannon entropy of one-particle and two-particle distributions as quantifiers of delocalization, in position ($S_{\Gamma }$) and momentum ($S_{\Pi }$) spaces, of the electron cloud in atomic systems. The results have been discussed on the basis of the atomic shell structure [41,60] and also in terms of relevant physical properties, such as, e.g., the atomic ionization potential (AIP) [41]. A summary of previous conclusions, and some additional ones, is given below.

In Ref. [60], the study was performed for the set of neutral atoms with number of electrons N≤36. There the main conclusions were as follows.

- Entropies display monotonic trends with *N*: decreasing for $S_{\Gamma }$, increasing for $S_{\Pi }$;
- Both entropic curves are structured accordingly with the presence of noble gases and systems with a semi or completely filled *d* valence subshell;
- The structure of position space curve is more prominent, but the interval the entropy values belong to is wider in momentum space.

A more recent work [41] has extended the above study in two different ways:

1. By considering a much larger set of neutral systems, up to N≤103;
2. By including ionized systems, particularly singly charged anions and cations with N≤54.

**Figure 1:**
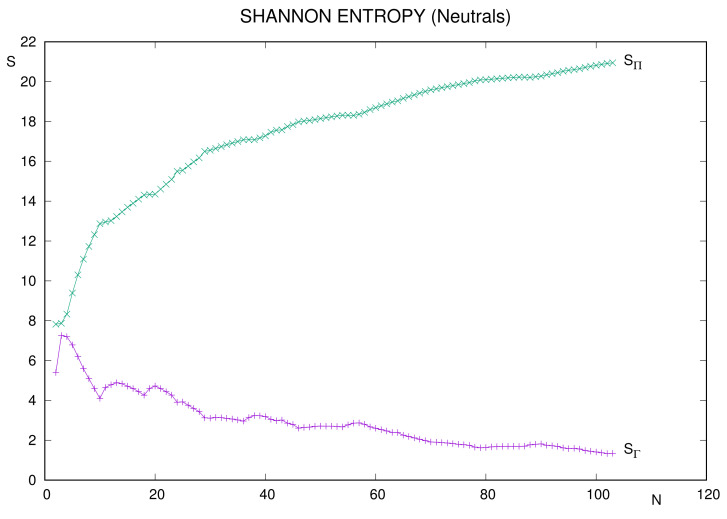
Two-electron Shannon entropies in position ($S_{\Gamma }$) and momentum ($S_{\Pi }$) spaces, for neutral atoms with number of electrons N=2–103. Atomic units (a.u.) are used.

In most cases, conclusions of the pioneering work remain valid. Regarding neutral systems, curves in Figure 1 display, overall, the respective monotonic behaviors. Moreover, previous comments on structural prominency and ranges of values apply now.

A detailed analysis of occurrence of extrema provides different results in each space. Local minima for position space entropy $S_{\Gamma }$ of neutral atoms correspond to the following:

- Closed shell systems (noble gases): N=10,18,36,54,86;
- Semi or completely filled *d* valence subshell: N=24,30,42,46,79;
- Similarly with 5f orbital: N=95,102.

No local extrema were referred in momentum space for the limited set N≤36 [60] of neutral atoms. The only ones displayed in Figure 1 occur beyond that threshold, the minima being N=38,56,88. All three are systems with filled valence subshell ns.

**Figure 2:**
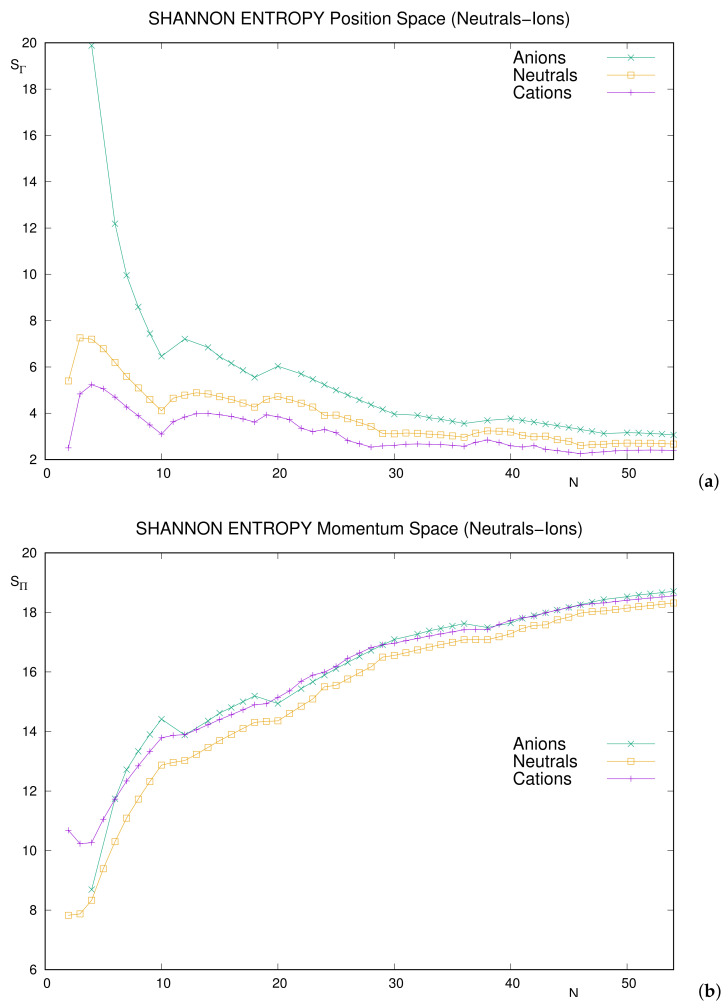
Two-electron Shannon entropies for neutral atoms and singly charged ions with number of electrons N≤54 in (**a**) position space ($S_{\Gamma }$) and (**b**) momentum space ($S_{\Pi }$). Atomic units (a.u.) are used.

Figure 2 provides joint results for neutrals and ions, particularly singly charged cations and anions. In all cases, systems with N≤54 are considered. A comparative analysis of curves, for each space, should be interpreted as follows: once fixed *N*, ordering of curves provides information about how the entropy varies as far as the nuclear charge *Z* moves to Z±1.

The most relevant comments are as follows.

- Ordering: In position space, the inequalities S(catio)<S(neutral)<S(anion) hold for arbitrary *N*. This means that increasing the nuclear charge (for fixed number of electrons) makes the entropy decrease due to the contraction of the electron cloud towards the nucleus.
  In momentum space, a not-so-strict inequality is verified, namely S(neutral)<S(ion). Thus, modifying the nuclear charge of the neutral in any manner makes the dispersion of speeds increase.
- Local extrema: All noble gases are displayed as local minima in position space for both cations and anions. However, they appear as local maxima for anions in momentum space. The momentum curve for cations is roughly increasing from N=3 onward. As for neutrals, the only (slight) minimum within this range is N=38.
  Few additional slight extrema appear in different curves.

It is worthy to remark that position–space curves are much more structured than the momentum–space ones, at least for neutrals (as emphasized in Ref. [60]) and cations. However, differences are not so prominent for anions, except the exchange of the roles of maximum–minimum for noble gases in passing from a space to the conjugate one. Justifying these different features for anions remains as an open question.

### 3.2. Mutual Information

In multielectronic systems, the electron pair density $\Gamma ({\vec{r}}_{1},{\vec{r}}_{2})$ in position space represents (properly normalized) the probability distribution of finding any pair of electrons at respective locations $\left({\vec{r}}_{1},{\vec{r}}_{2}\right)$. Obviously, for uncorrelated electron systems, the pair distribution is merely a product of one-electron distributions $\rho ({\vec{r}}_{1})$ and $\rho ({\vec{r}}_{2})$ by considering the indistinguishability of electrons.

The same reasoning applies in momentum space, dealing with the pair distribution $\Pi ({\vec{p}}_{1},{\vec{p}}_{2})$ and the respective one-electron functions $\gamma ({\vec{p}}_{1})$ and $\gamma ({\vec{p}}_{2})$. This means that, in both spaces, determining electron correlation is equivalent to measuring differences between the electron pair distribution in a given space and the product of one-electron distributions in the same space.

Those differences have been studied in the past, by considering the so-called ‘mutual information’ (*I*) between the respective pairs of variables in each conjugate space for some specific atomic systems. A pioneering study is included in Ref. [60] for neutrals atoms with a number of electrons up to N=36, described in terms of the near Hartree–Fock wavefunctions of Clementi and Roetti [66]. The present section provides an expanded study of mutual information *I* in atoms, reaching up to N=103 and also including ionized systems. In performing this, the posterior wavefunctions of Koga [62,63] are employed. Some of the known conclusions remain, but others are modified.

#### 3.2.1. Mutual Information of Neutral Atoms

**Figure 3:**
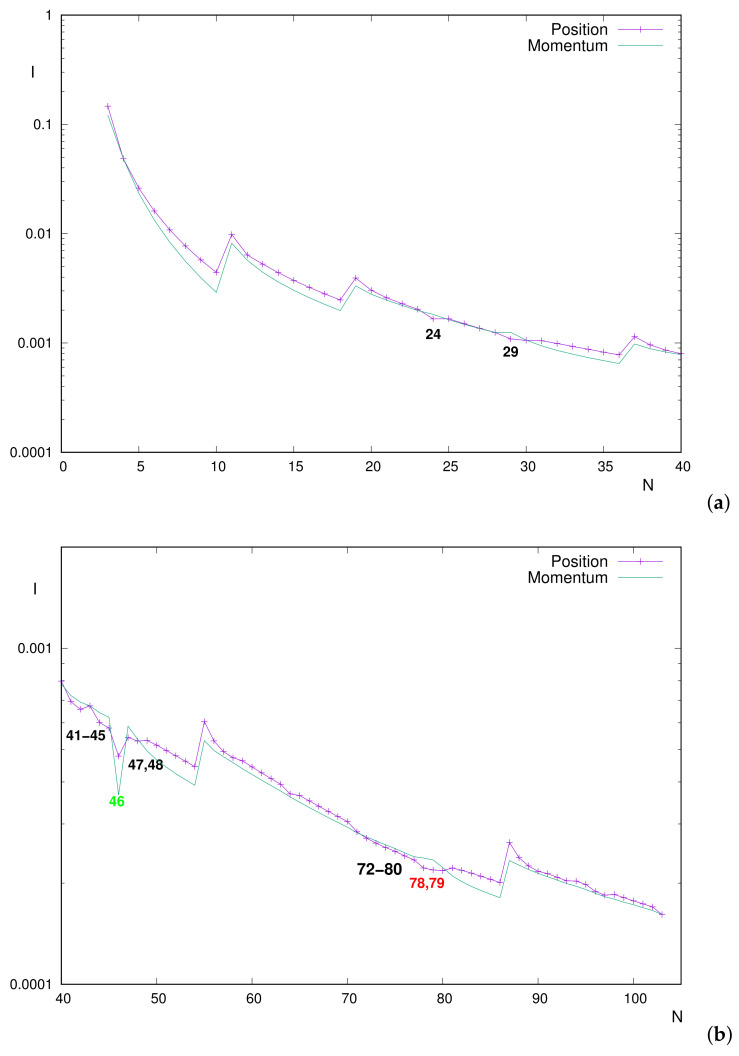
Mutual information *I* in position and momentum spaces for neutral atoms with number of electrons (**a**) N≤40 and (**b**) N=40–103. Atomic units (a.u.) are used.

Let us remind the reader that, for neutral atoms, the nuclear charge (in a.u.) and the number of electrons are identical, i.e., Z=N. The numerical results displayed in Figure 3a, namely the *I* values for N=3–40, include the aforementioned range N≤36. The curves correspond to mutual information in position ($I_{r}$) and momentum ($I_{p}$) spaces.

Main conclusions derived in previous studies, particularly by Sagar et al. [60], are as follows:

1. The global decreasing trends of both $I_{r}$ and $I_{p}$;
2. The marked peaks (minima) at noble gas atoms, as systems with the smallest value within a period;
3. The inequality $I_{r}>I_{p}$ along the entire range, with a few exceptions: particularly for N=24,29, both with anomalous shell-filling and 3d outermost orbital (half-filled for N=24, completely filled for N=29).

The above conclusions for N≤36 are fully ratified in the present work. The mentioned ordering $I_{r}>I_{p}$ is clearly appreciated in the figure, as well as the exceptions N=24 and N=29, with respective differences 10% and 15% between position/momentum values. This means that, for most neutral atoms, the electron correlation is higher in position space than in momentum space.

An additional exception, found here but not reported before, is N=30. However, in this case, $I_{r}$ and $I_{p}$ are almost identical, with differences below 0.4%. Notice that different wavefunctions were employed in the respective works [62,66], possibly as justification of the ‘opposite ordering’ for N=30, an additional system with completely filled 3d subshell (as is also N=29).

In proceeding beyond N=36, other ‘exceptions’ (i.e., systems with $I_{p}>I_{r}$) are found, as observed in Figure 3b. The inverse inequality now holds for many systems; thus, perhaps the term ‘exception’ becomes inappropriate. They are grouped into two blocks: N=41–48 (excepting N=46) and N=72–80. Once again, an additional system (N=103) verifies the inequality but with extremely small position/momentum differences, below 0.1% (thus, they are completely indistinguishable in the figure and almost numerically as well).

- The first group contains all neutral atoms in the periodic table with half-filled inner 5s subshell (N=41–45,47) and the closed-subshells one N=48 with valence orbital 4d (the others have the same valence orbital, completely filled only for N=47 as for N=48).
  For this atomic set, $I_{r}-I_{p}$ differences do not reach 8%, in some cases below 1%; thus, they are not so prominent as for the well-known N=24,29 discrepancies.
  It is worthy to remark that N=46, staying out of this set, is the unique system (within this range and in the entire periodic table as well) with completely empty inner subshell 5s, which is what distinguishes it from others.
- The set N=72–80 is characterized for the filling of the 5d valence orbital. Two systems are remarkable: N=78,79 are the only ones with half-filled inner 6s subshell, and they are emphasized in the figure. Their position–momentum deviations are clearly appreciated, rounding 7% of numerical differences, remaining below 2% for all the others.
- As for the range N≤40 in the previous figure, marked minima are displayed again for noble gases, namely N=54,86. Thus, it is concluded that, almost systematically, both $I_{r}$ and $I_{p}$ decrease along a given period and notably increase as a new period begins. Apart from the slight extrema N=24,29, the additional marked minimum N=46 in both spaces is found. Thus, this system ‘divides’ the monotonic decrease along 4d into two pieces (including, respectively, atoms with completely filled valence orbital or not).

We could say that, overall, conclusions derived in Ref. [60] regarding structure and ordering of $I_{r}$ and $I_{p}$ for neutral atoms within the range N=3–36 are here extended up to N=103. Perhaps the most relevant differences are as follows: (i) the existence of additional systems with higher correlation in momentum space than in position space, once again associated with the filling of *d* valence subshells; and (ii) the prominent local minimum N=46, in both spaces, in addition to those of noble gases.

#### 3.2.2. Mutual Information of Singly Charged Ions

Ionization of a neutral system is achieved by breaking the equality N=Z between the number of electrons and nuclear charge. Thus, for atomic ions, it is necessary to choose one of the above variables to analyze the corresponding dependence of the mutual information on it.

Attending to the preceeding results in this work and their interpretation, it appears preferable to deal with *N* as the characterizer of the different ground-state systems, because its value determines their shell structure.

**Figure 4:**
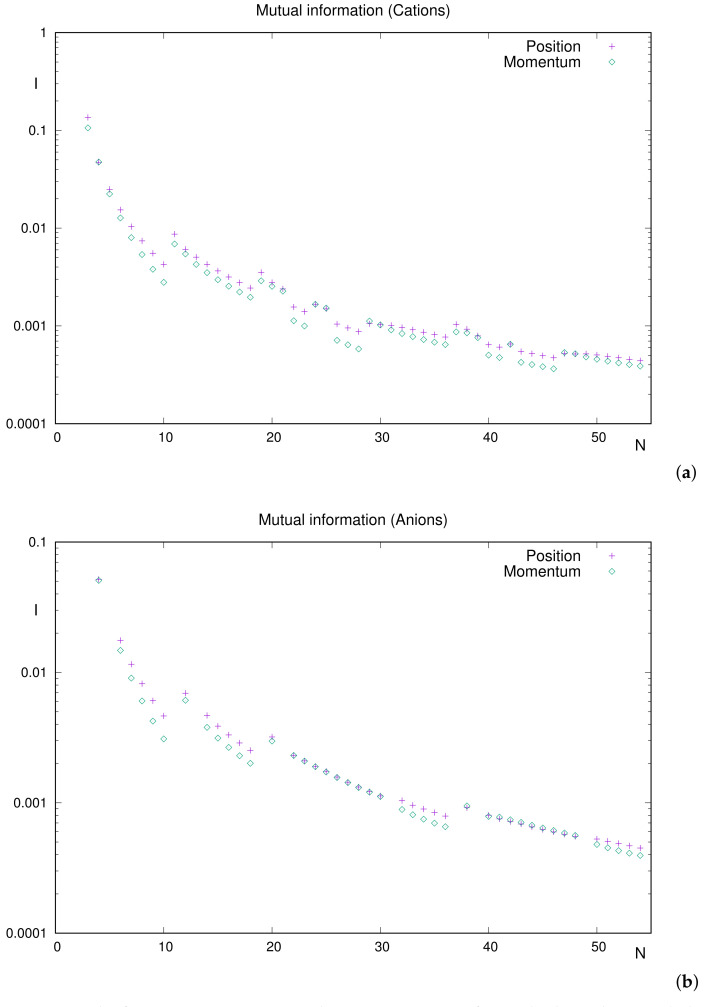
Mutual information *I*, in position and momentum spaces, for singly charged ions with the number of electrons N≤54: (**a**) cations and (**b**) anions. Atomic units (a.u.) are used.

Figure 4 displays $I_{r}$ and $I_{p}$ for both cations and anions. It is appropriate to perform a study of structure and ordering of curves. In Figure 4a, for cations, the global decreasing trend of both piece-wise curves is observed, almost systematically with the position one above the momentum one (accordingly with the inequality $I_{r}>I_{p}$).

A detailed analysis shows us the following:

- Relevant extrema correspond to the following: (i) local minima at N=10,18,36,46,54 (i.e., noble gases together with the anomalous N=46) and (ii) local maxima at N=24,29,42. Let us emphasize that N=42 possesses half-filled valence subshell 4d.
- The above extrema are common to both $I_{r}$ and $I_{p}$. An extremely slight one (N=48) occurs only in position space.
- Most cations verify $I_{r}>I_{p}$. The scarce and slight exceptions are (position/momentum differences in parentheses): N=4(0.7%), N=25(2%), N=29(7%), N=47(3%) and N=48(0.5%). Maybe N=29 is the only one observed (hardly) in the figure.

The above discussion on cations is straightforwardly applied to anions, Figure 4b. The analysis on structure becomes simpler (consider the absence, in the list of available anions, of systems with a unique electron at the outermost subhell). A similar situation happens with ordering due to the well-delimited set of atoms with higher mutual information in momentum space:

- The only local minima, of both $I_{r}$ and $I_{p}$, are noble gases (N=10,18,36,54). We could say that the curve consists of four well-defined pieces (except for the ‘gaps’ due to the aforementioned absences), corresponding to the respective periods.
- The exceptions to rule $I_{r}>I_{p}$ are as follows: N=38,41–48. Notice that, apart from N=38, the rest of the systems correspond to the filling of the 4d subshell. Position/momentum differences round to 2–3% in all cases.

Summarizing the results for ions, it is observed that (i) less correlated systems include noble gases systematically, in both spaces, and (ii) the correlation is usually higher in position space, with some exceptions associated to the filling of *d* subshells and with very slight predominance of $I_{p}$ over $I_{r}$.

### 3.3. Quantum Similarity

A different method of comparing the position–space distributions $\Gamma ({\vec{r}}_{1},{\vec{r}}_{2})$ and $\rho \left({\vec{r}}_{1}\right)\rho \left({\vec{r}}_{2}\right)$, and similarly in momentum space, is to determine the quantum similarity among them. This is performed quantitatively in terms of their QSI, which provides a measure of the overlapping among both distributions. Both $QSI_{r}$ and $QSI_{p}$ are displayed as functions of the number of electrons *N* for neutral atoms with N≤103.

**Figure 5:**
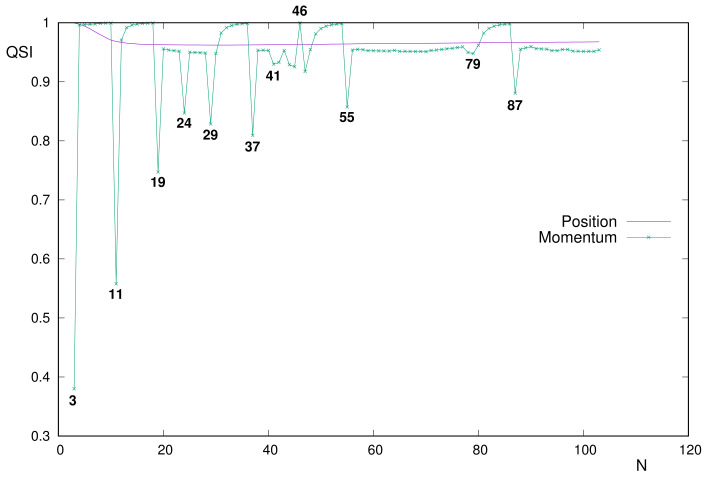
Quantum Similarity Index QSI in position and momentum spaces, between the two-electron distribution and the product of one-electron distributions for a neutral atoms with number of electrons *N* = 3–103.

The most prominent feature displayed in Figure 5 is the extremely different structure of the respective curves for position and momentum spaces: the former being roughly monotonic and the latter with numerous extrema, with many of them being very apparent.

#### 3.3.1. Neutral Atoms: Position Space

**Figure 6:**
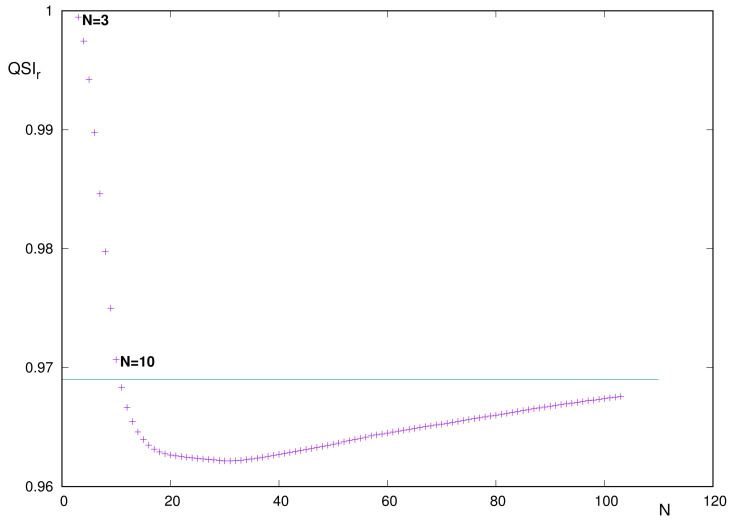
Quantum Similarity Index $QSI_{r}\equiv QSI\left(\Gamma ,\rho_{1}\rho_{2}\right)$ in position space, between the two-electron distribution and the product of one-electron distributions, for neutral atoms with number of electrons *N* = 3–103.

The curve $QSI_{r}$ of similarity in position space demands a zoomed display because of the absence of structure and the extremely narrow interval of values, at least from N∼10 onward. These features are clearly appreciated in Figure 6.

A careful reading of the data suggests the following comments:

- The curve is unimodal, with absolute minimum $QSI_{r}=0.962157$ at N=31.
- The starting and ending heights are $QSI_{r}=0.999459$ (N=3) and $QSI_{r}=0.967576$ (N=103), respectively.
- From the above, it is concluded that $QSI_{r}\in \left[0,9621,0.9995\right]$ for the entire set N=3–103.
- As an indication, let us notice that $QSI_{r}=0.9683$ for N=11, a value close to that for the right-hand-side extremum N=103. This means that, for the range N=11–103, the similarity value belongs to the narrow interval $0.962<QSI_{r}<0.969$. Consequently, the only systems with $QSI_{r}\geq 0.97$ correspond to 3≤N≤10.

The above comments indicate that, for reasons to be specified, $QSI_{r}$ progressively decreases for very light systems, until reaching a roughly constant value for the rest of the Periodic Table.

**Figure 7:**
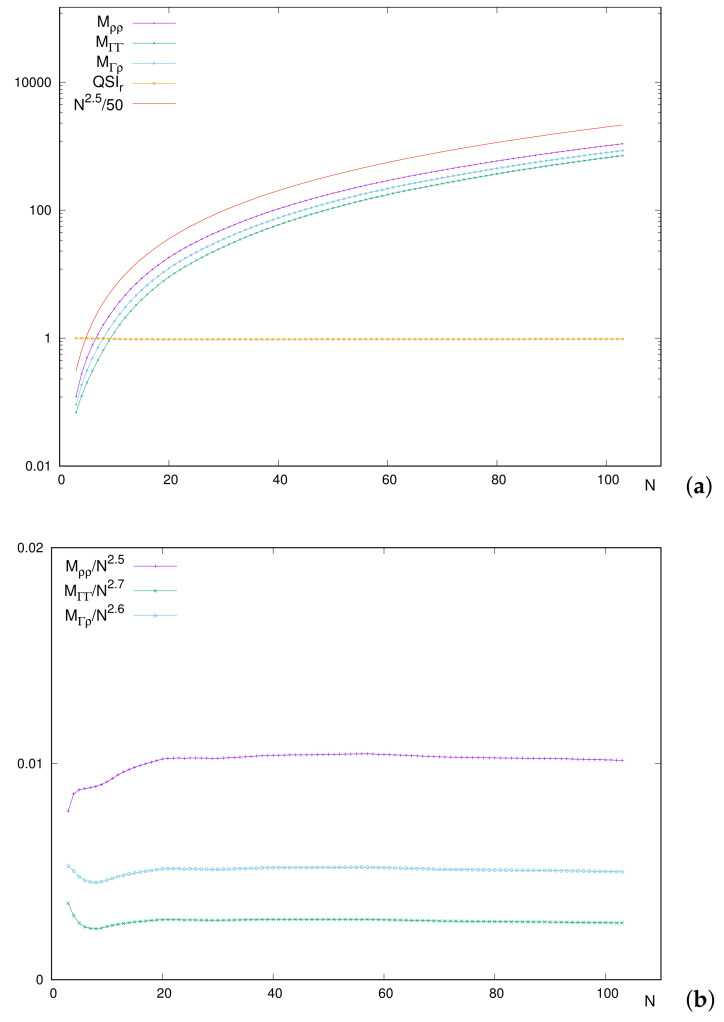
(**a**) Integrals defining $QSI_{r}\equiv M_{\Gamma \rho }/{\left(M_{\rho \rho }M_{\Gamma \Gamma }\right)}^{1/2}$, and (**b**) integrals scaled with powers of *N* for neutral atoms with number of electrons N=3–103. Atomic units (a.u.) are used.

Looking for the origin of the different behaviors of $QSI_{r}$ for short and long-*N* values, it is worthy to remember that the definition of the Quantum Similarity Index QSI includes three terms: the overlap measure $QSM(\Gamma \left({\vec{r}}_{1},{\vec{r}}_{2}\right),\rho \left({\vec{r}}_{1}\right)\rho \left({\vec{r}}_{2}\right))$ in the numerator (to be denoted as $M_{\Gamma \rho }$) and the respective ‘disequilibria’ within the square root at the denominator (with simplified notations $M_{\rho \rho }$ and $M_{\Gamma \Gamma }$). Let us explore separately the behavior of each integral defining $QSI_{r}$, as is performed in Figure 7.

First, Figure 7a includes the curves for each integral, together with (i) $QSI_{r}$, and (ii) an orientative power-like curve to estimate the *N*-dependence of the integrals. As expected, $QSI_{r}$ is displayed as a roughly straight line of height close to one. The similar shape of curves for integrals and the power-like one is observed with soft and monotonically increasing paths. Let us emphasize that the power $N^{5/2}$ is only provided for a visual orientation, and it is not the result of any numerical treatment.

Having in mind that an exact dependence on *N*, identical and separable for each integral, would provide a constant $QSI_{r}$ (which is not the case), we feel enforced to perform a more detailed analysis of such a dependence. Figure 7b provides the relative behaviors with respect to some power of *N*, by means of quotients as $M/N^{\alpha }$. In this sense, a given integral *M* with a dependence of proportionality to $N^{\alpha }$ would provide a constant quotient, displayed as a horizontal line at some height.

Again for orientative powers, scaled curves in Figure 7b are roughly constant for a wide range of *N*, estimatedly for N≥20. Notice that the chosen powers imply the invariance of $QSI_{r}$ for constant quotients. This means that including a factor $1/N^{2.6}$ in the numerator of QSI, and the two factors $1/N^{2.5}$ and $1/N^{2.7}$ within the square root in denominator, keeps the value of QSI unchanged. Thus, the analyses of $QSI_{r}$ are equivalent, including the mentioned factors.

Focusing now on the region of light systems, some differences are appreciated. The main one corresponds to the deviation from the respective constant values. However, additionally, it is observed that the respective deviations occur at different places and with different amplitudes. Most probably, this causes the ‘global deviation’ of $QSI_{r}$ from the constant value reached for heavier systems.

What is especially remarkable is the unimodal shape of $M_{\Gamma \rho }$ and $M_{\Gamma \Gamma }$ in the region of low *N*, while $M_{\rho \rho }$ maintains an increasing behavior. Let us remember that $M_{\rho \rho }$ is included at the denominator of $QSI_{r}$ so that the different and increasing behavior of $M_{\rho \rho }$ in that region provokes a decrease in $QSI_{r}$, as previously displayed in Figure 6. Thus, it is concluded that the integral containing only the uncorrelated distribution $\rho \left({\vec{r}}_{1}\right)\rho \left({\vec{r}}_{2}\right)$ is responsible for the decrease in $QSI_{r}$ for low *N*, once compared to the two integrals including the correlated function $\Gamma ({\vec{r}}_{1},{\vec{r}}_{2})$.

#### 3.3.2. Neutral Atoms: Momentum Space

Local minima of the momentum similarity $QSI_{p}$ are clearly appreciated in Figure 5 for all alkalines, the only systems below the value 0.9, together with the pair of anomalous N=24,29, as will be discussed below.

On the opposite, the highest $QSI_{p}$ is reached for systems with *p* valence subshell, belonging to the narrow interval $QSI_{p}\in \left[0.981,0.9995\right]$. Moreover, alkaline-earth N=4 with $QSI_{p}=0.996$ belongs to it, and the most uncorrelated system N=46 is the unique one above that interval ($QSI_{p}=0.99990$). Other alkaline-earths are within the values of $0.953<QSI_{p}<0.971$.

Before continuing the discussion in detail, let us observe that most heavier systems (namely, N=56–103 except the aforementioned alkalines and alkaline-earths and those filling the 6p subshell) display extremely similar values. In fact, inequalities $0.951<QSI_{p}<0.962$ apply for them, with only two exceptions: N=78,79 is displayed slightly below, with respective similarities 0.950 and 0.948. Their anomalies in shell-filling (i.e., half-filled inner 6s subshell) are revealed along the $QSI_{p}$ curve in the figure.

The analysis of systems with *d* valence subshells appears, in some cases, more difficult because it requires considering the occupation of inner subshells. For systems N=21–30 with outermost 3d subshell, the presence of the above-mentioned apparent peaks N=24,29 is due to the promotion of an electron from the inner 4s. Thus, they deviate notably from the range [0.948,0.954] to respective minima 0.847 and 0.829.

For the atomic set N=39–48 corresponding to the filling of 4d, it is necessary to distinguish different cases. As remarked above, N=46 is especially relevant due to its highest uncorrelation and because it is the unique system with a inner 5s subshell that is completely empty. On the other hand, for atoms on that set with 5s completely filled the $QSI_{p}$ belongs to [0.953,0.955]. Finally, for anomalous systems with semi-filled 5s, the similarities are as low as $0.917<QSI_{p}<0.933$, with the unique exception N=43 (0.953, as for non-anomalous 5s4d).

**Figure 8:**
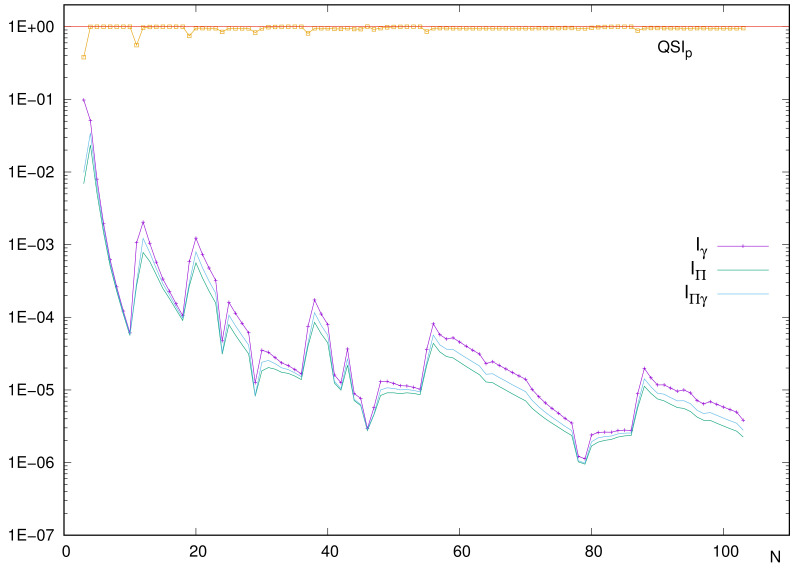
Integrals defining $QSI_{p}\equiv M_{\Pi \gamma }/{\left(M_{\gamma \gamma }M_{\Pi \Pi }\right)}^{1/2}$, for neutral atoms with number of electrons N=3–103. Atomic units (a.u.) are used.

Here, again a study of the integral components is addressed. As was performed in the position case, we adopt the simplified notation $M_{\Pi \gamma }$, $M_{\Pi \Pi }$ and $M_{\gamma \gamma }$ for the respective momentum–space integrals, displayed in Figure 8. It is observed that (i) curves of the three integrals are very similar, appearing as ‘embedded’, and (ii) in overall, all integrals display local extrema at the positions where $QSI_{p}$ does.

Differences among curves do not consist merely on a shifting factor, which is what would produce a constant similarity. For illustration, $QSI_{p}$ varies remarkably in passing from a closed-shell system to an alkaline and similarly from this to an alkaline-earth. The reason is the different rate of change for the disequilibrium $M_{\gamma \gamma }$ of the one-particle density as compared to the other two integrals. There exist other examples: The ratios between the respective integrals in passing from N=3 to N=4 are 3.4 and 3.5 (for $M_{\Pi \Pi }$ and $M_{\Pi \gamma }$, respectively), while $M_{\gamma \gamma }$ decreases by a factor 0.5. For the marked peak N=46, the ratios are (2.2,2.2,2.6) when compared to its preceding neighbor N=45 and (1.6,1.6,1.9) with respect to its succeeding one N=47. In both cases, the value that differs corresponds to integral $M_{\gamma \gamma }$.

#### 3.3.3. Singly Charged Ions

The above study on correlation in neutral systems can be straightforwardly extended to ionized ones by using the corresponding Hartree–Fock wavefunctions provided in Ref. [62]. Their availability occurs for singly charged ions with N≤54 but, in the case of anions, excludes those with a unique electron in the outermost subshell.

**Figure 9:**
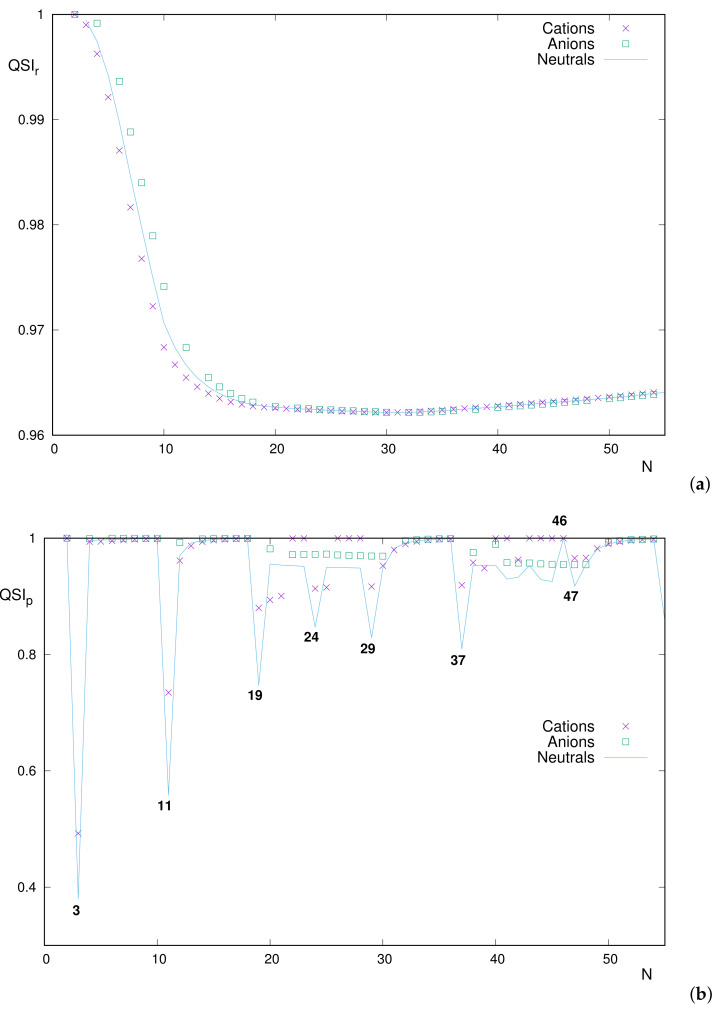
Quantum Similarity Index QSI between the two-electron distribution and the product of one-electron distributions, for neutral atoms and some singly charged ions with a number of electrons N≤54 in (**a**) position space ($QSI_{r}$) and (**b**) momentum space ($QSI_{p}$). Atomic units (a.u.) are used.

Figure 9 displays QSI among correlated and uncorrelated distributions for a given system, with a number of electrons *N*, in both conjugate spaces: position in Figure 9a and momentum in Figure 9b. This was performed for neutrals (i.e., atoms with nuclear charge Z=N), cations (Z=N+1) and anions (Z=N−1).

All position–space curves follow roughly the same path, as shown in Figure 9a. It is noticeable that, as for neutrals, the ionic curves are unimodal, and their absolute minimum is also characterized as the next one once the 3d subshell becomes completely filled. Therefore, the minimum occurs at N=31 for neutrals and cations and at N=32 for anions (let us remind the reader that N=31 is not included in the available set of anions).

Another interesting feature that is hardly observed in the figure for medium-heavier systems is the ordering of the values $QSI_{r}\left(cation\right)$, $QSI_{r}\left(neutral\right)$ and (if included) $QSI_{r}\left(anion\right)$, for a given *N* (i.e., the relative vertical positions of the curves Z=N and Z=N±1 at location *N*). In the following, the inequalities involving $QSI_{r}\left(anion\right)$ refer to the cases in which the anion is available, focusing only on the neutral-cation relationship in other cases.

Two orderings appear exactly:

(i) $QSI_{r}\left(anion\right)>QSI_{r}\left(neutral\right)>QSI_{r}\left(cation\right)$ for systems with N≤30;
(ii) The reversed one $QSI_{r}\left(anion\right)<QSI_{r}\left(neutral\right)<QSI_{r}\left(cation\right)$ for N>30.

The first ordering (displayed for lighter systems) implies that, keeping *N* fixed, similarity decreases as the nuclear charge *Z* increases. However, the opposite occurs for heavier systems with completely filled 3d subshells: Increasing *Z* produces correlated and uncorrelated distributions to augment their similarity. Perhaps surprisingly, the exchange between the neutral-anion curves occurs at exactly the same point as for the neutral-cation ones.

Therefore, it appears essential, for the study of position–space correlation based on similarity in neutral atoms and singly charged ions, to distinguish the cases in which a *d* subshell of the system is fulfilled or not.

**Figure 10:**
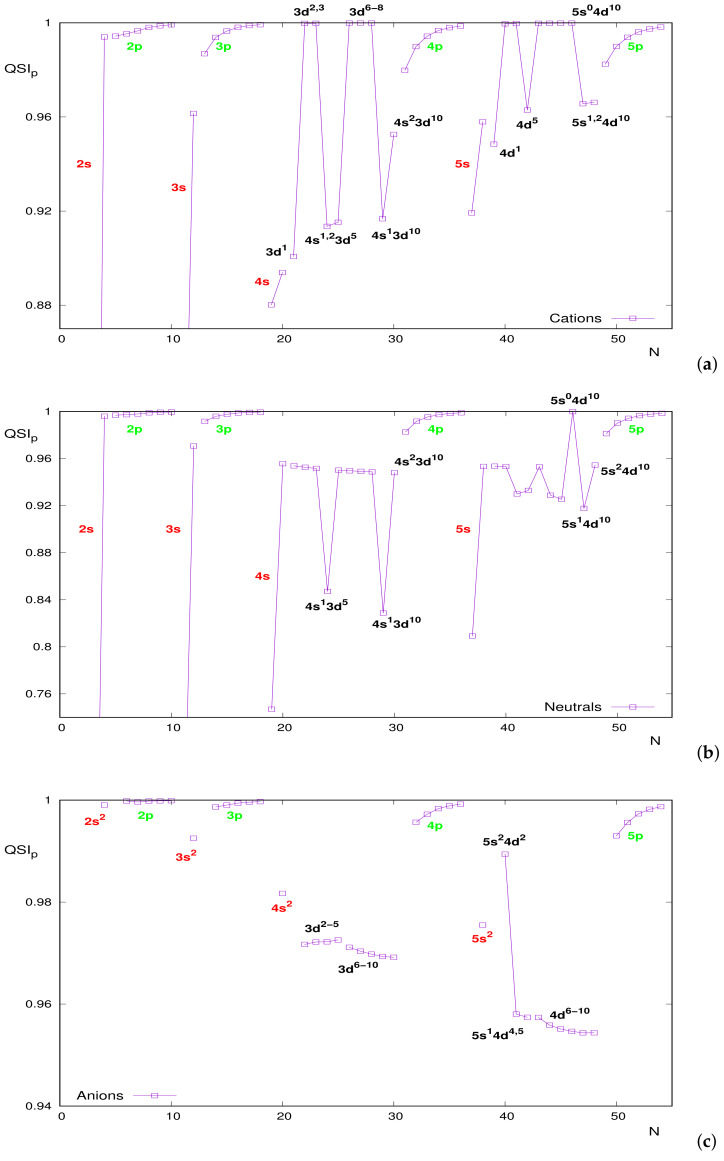
Quantum Similarity Index $QSI_{p}$ in momentum space between the two-electron distribution and the product of one-electron distributions for atomic systems with a number of electrons N≤54: (**a**) cations, (**b**) neutrals and (**c**) anions.

A similar analysis in momentum space is more difficult, as observed in Figure 9b. However, in spite of the absence of a systematic ordering, the relevance of atomic shell-filling for the interpretation of momentum curves is observed. This is more clearly appreciated in Figure 10, where $QSI_{p}$ for the three atomic species (cations, neutrals and anions) is drawn separately into respective figures. To visualize more clearly the structural patterns, an appropriate vertical range has been chosen in spite of leaving out a couple of systems (the previous Figure 9b encloses all of them).

Maybe the most relevant feature, common to the three figures, is the specific behavior along different types of subshells and, at times, the global tendency of a given type. Some comments are in order:

- Lines of high slope are displayed for the transitions from systems with half-filled external *s* subshell to completely filled ones, thus with a relevant loss of correlation. This applies to neutrals and cations (the anions set does not include the half-filled case). A global decreasing tendency is observed in the three figures for both *s*-filling cases.
- If the valence subshell is a np one, values are close to unity and quite close for lighter systems. Along a given *p* subshell, an increasing behavior is observed, roughly displayed for low *n* in anions.
- Valence subshells 3d and 4d require a more detailed analysis. Figure 10a for cations displays prominent minima at *N* corresponding to the cases of *d* subshell (i) with a unique electron, (ii) half-filled or (iii) completely filled. These two filling cases are clearly appreciated also in Figure 10b for neutrals. Additionally, both for cations and neutrals, it appears that there is a clearly distinguished N=46 corresponding to the unique system with empty internal 5s subshell (structure $5s^{0}4d^{10}$), with $QSI_{p}$ roughly unity. As soon as an electron is added, similarity decreases markedly.
- Regarding anions, in Figure 10c, the half-filled case in the 3d subshell marks a change of trend, from increase to decrease. A change of decreasing rate is observed in 4d, after a very apparent fall in passing from the inner $5s^{2}$ to $5s^{1}$.

In summary, the structural features of $QSI_{p}$ are very similar for the three atomic species with *s* or *p* valence subshells, but differences among them are much more noticeable in the case of *d* valence subshells.

## 4. Conclusions

Mutual information, *I*, and Quantum Similarity Index, QSI, have been applied as comparative functionals, for a large set of many-electron systems, by means of their respective one-electron and two-electron distributions. The systems considered include both neutral atoms (N=Z) and singly charged ions (N=Z±1). An in-depth study was performed by considering (i) both position/momentum conjugate spaces and (ii) neutral systems with a nuclear charge up to Z=103 and singly charged ions (cations and anions) with a number of electrons as far as N=54. The differences between the monoelectronic and the electron pair densities can be interpreted as a measure of the electron correlation of the system. This means that, in both spaces, determining electron correlation is equivalent to measuring differences between the electron pair distribution in a given space and the product of one-electron distributions in the same space.

Those differences, in terms of mutual information *I*, have been studied in the past [60] for neutrals atoms with a number of electrons up to N=36. The present work provides an expanded study, reaching up to N=103 and also including ionized systems. In performing this, the posterior wavefunctions of Koga are employed. We could say that, overall, the conclusions derived from that previous work regarding structure and ordering of $I_{r}$ and $I_{p}$ for neutral atoms within the range N=3–36 are here extended up to N=103. Perhaps the most relevant differences are as follows: (i) the existence of additional systems with higher correlation in momentum space than in position space, once again associated with the filling of *d* valence subshells; and (ii) the prominent local minimum N=46, in both spaces, in addition to those of noble gases. Summarizing the results for ions, it is observed that (i) less correlated systems include noble gases systematically, in both spaces, and (ii) correlation is usually higher in position space, with some exceptions associated to the filling of *d* subshells and with very slight predominance of $I_{p}$ over $I_{r}$.

A similar analysis has been performed in terms of the Quantum Similarity Index, QSI. For neutral atoms, the curve $QSI_{r}$ of similarity in position space is characterized by the absence of structure and the extremely narrow interval of values. The $QSI_{r}$ curve is unimodal and belongs to the narrow interval 0.962–0.969 for most systems, except for the lighter ones N≤10. In momentum space, $QSI_{p}$ has a richer structure, with apparent local minima for all alkalines, together with the pair of anomalous systems N=24,29.

The study on correlation in neutral systems has been straightforwardly extended to ionized ones (anions and cations). All position–space curves (neutrals and ions) follow roughly the same path. It is noticeable that, as for neutrals, the ionic curves are unimodal, their absolute minimum determined also by the filling of the 3d subshell. Another interesting feature is the ordering, for fixed *N*, of the values for the three atomic species: For lighter systems, similarity decreases as the nuclear charge *Z* increases. However, the opposite occurs for heavier systems, i.e., increasing *Z* produces correlated and uncorrelated distributions that augment their similarity. In spite of the absence of a systematic ordering, the relevance of atomic shell-filling for the interpretation of the curves in momentum space is observed. The structural features of $QSI_{p}$ are very similar for the three atomic species with *s* or *p* valence subshells, but differences among them are much more noticeable in the case of *d* valence subshells.
